# Evolution of BPSD and psychotropic drug therapy in patients with dementia

**DOI:** 10.1002/alz.091229

**Published:** 2025-01-03

**Authors:** Rosanna Pullia, Sara Rogani, Maria Giovanna Bianco, Irene Taverni, Noemi Pardini, Leonarda Maltese, Andrea Giusti, Valeria Calsolaro, Agostino Virdis

**Affiliations:** ^1^ Azienda Ospedaliero Universitaria Pisana, Pisa, PI Italy

## Abstract

**Background:**

Despite being off‐label, antipsychotics and antidepressants are widely prescribed to manage Behavioural and Psychological Symptoms of Dementia (BPSD). Aim of our study was to assess the evolution of BPSD and psychoactive pharmacological therapy in older patients with dementia.

**Method:**

In this retrospective observational study, outpatients with dementia underwent a Comprehensive Geriatric Assessment (ADL, IADL, CFS and CIRS), at baseline, 6‐month and 12‐month follow‐up visits. Additionally, cognitive impairment (MMSE) and severity of BPSD (NPI) were determined.

**Result:**

266 patients (mean age 82.69 ± 10.19; 69.58% female) were enrolled; 169 underwent a 6‐month follow‐up and 111 had a 12‐month follow‐up. We observed a progressive decline in MMSE [18.89 (±5.02), 17.69 (±5.72), 17.25 (±5.70); p<0.001], ADL [5 (±3), 4 (±3), 4 (±3); p<0.001], and CFS [5.44 (± 1.23), 5.67 (±1.78), 5.77 (±1.15): p<0.001]. No differences were found in the prescription of antipsychotics over 12 months. However, there was a notable increase in Trazodone prescription between baseline and 6‐month follow‐up (18.42% at baseline, 31.55% at six months, p>0.001), with consistent prescription rate at 12 months (32.43%). Among the neuropsychiatric symptoms (anxiety, agitation, sleep disturbances), there was a significant increase in agitation at the 6‐month follow‐up compared to baseline (p = 0.026). The prescription of antidepressants significantly increased between baseline and 6‐month follow‐up (p = 0.003), without a significant increase in symptoms like depression, anxiety, or eating behaviour disorders. Patients taking antidepressants at baseline were mostly female (p = 0.043), didn’t significantly differ in age and NPI scores both at baseline and six months, but showed lower frailty levels compared to non‐users (p = 0.009), higher prevalence of anxiety (p = 0.015), appetite disturbance (p = 0.017) and depression (p = 0.034). Antidepressants were more frequently taken by women (p = 0.03) and linked to clear depressive symptoms both at 6‐month (p<0.001) and 12‐month follow‐up (p = 0.025), with higher burden of psychiatric symptoms observed at six months and baseline.

**Conclusion:**

In older patients with dementia, BPSD gradually increased, together with functional and cognitive decline over 12 months. Managing agitation led to an increased prescription of trazodone, while antidepressant medications were used in follow‐ups for depressive symptoms; more importantly, prescription of antipsychotics did not increase, aligning with recommendations regarding their safety profile.

